# Efficacy and safety of upadacitinib over 84 weeks in Japanese patients with rheumatoid arthritis (SELECT-SUNRISE)

**DOI:** 10.1186/s13075-020-02387-6

**Published:** 2021-01-06

**Authors:** Hideto Kameda, Tsutomu Takeuchi, Kunihiro Yamaoka, Motohiro Oribe, Mitsuhiro Kawano, Masayuki Yokoyama, Aileen L. Pangan, Yuko Konishi, Sebastian Meerwein, Yoshiya Tanaka

**Affiliations:** 1 Division of Rheumatology, Department of Internal Medicine, Faculty of Medicine, Toho University, 2-22-36, Ohashi, Meguro-ku, Tokyo, 153-8515 Japan; 2 Keio University School of Medicine, 2 Chome-15-45 Mita, Minato City, Tokyo, 108-8345 Japan; 3 Kitasato University School of Medicine, 1 Chome-15-1 Kitazato, Minami Ward, Sagamihara, Kanagawa 252-0374 Japan; 4 Department of Internal Medicine, Oribe Clinic of Rheumatism and Medicine, Oita Oita-shi Otemachi 2-1-15, Oita, Japan; 5 Rheumatology, Honjo Rheumatism Clinic, Takaoka, Toyama 933-0874 Japan; 6 Immunology, AbbVie GK, 16F, 3 Chome-1-14F Shibaura, Minato City, Tokyo, 105-0023 Japan; 7 Immunology, AbbVie, 1400 Sheridan Rd, North Chicago, IL 60064 USA; 8 Pharmaceutical Development, AbbVie Deutschland GmbH & Co KG, Knollstraße 50, 67061 Ludwigshafen am Rhein, Germany; 9 The First Department of Internal Medicine, University of Occupational and Environmental Health, 1-1 Iseigaoka, Yahatanishi Ward, Kitakyushu, Fukuoka 807-8555 Japan

**Keywords:** Janus kinase inhibitor, Japanese, Rheumatoid arthritis, Upadacitinib

## Abstract

**Background:**

The objective of the study was to evaluate the efficacy and safety of upadacitinib over 84 weeks in Japanese patients with active rheumatoid arthritis (RA) and an inadequate response to conventional synthetic disease-modifying anti-rheumatic drugs.

**Methods:**

All patients completing a 12-week, randomized, double-blind treatment period entered a blinded extension and continued upadacitinib 7.5, 15, or 30 mg once daily (QD), or were switched from placebo to upadacitinib 7.5, 15, or 30 mg QD. Efficacy and safety were assessed over 84 weeks.

**Results:**

Of 197 randomized patients, 187 (94.9%) completed the 12-week period and entered the blinded extension; 152 (77.2%) patients were ongoing at week 84. At week 84, the proportions of patients achieving a 20% improvement in American College of Rheumatology criteria (ACR20) were 85.7%, 77.6%, and 58.0% with continued upadacitinib 7.5, 15, and 30 mg, respectively (nonresponder imputation), and were similar in patients who had switched from placebo. Favorable response rates were also observed for more stringent measures of response (ACR50/70) and remission (defined by the Disease Activity Score of 28 joints with C-reactive protein, Clinical Disease Activity Index, or Simplified Disease Activity Index). The 15 mg and 30 mg doses of upadacitinib were associated with more rapid and numerically higher initial responses for some measures of disease activity and remission compared with the 7.5 mg dose. Rates of adverse events, infection, opportunistic infection, serious infection, and herpes zoster were lower with upadacitinib 7.5 and 15 mg versus 30 mg.

**Conclusions:**

Upadacitinib demonstrated sustained efficacy and was well tolerated over 84 weeks in Japanese patients with RA, with upadacitinib 15 mg offering the most favorable benefit–risk profile.

**Trial registration:**

ClinicalTrials.gov NCT02720523. Registered on March 22, 2016.

**Supplementary Information:**

The online version contains supplementary material available at 10.1186/s13075-020-02387-6.

## Background

The Janus kinase (JAK) family (JAK1, 2, 3, and tyrosine kinase 2 [TYK2]) are important mediators of multiple cytokine-signaling pathways involved in normal cellular processes, as well as in the pathogenesis of rheumatoid arthritis (RA) and other immune-mediated inflammatory diseases [1–3]. Orally administered JAK inhibitors belong to the class of targeted synthetic disease-modifying anti-rheumatic drugs (DMARDs) and are currently recommended globally as treatment options for patients with RA who have moderate or high disease activity despite conventional synthetic DMARD (csDMARD) therapy, or in patients who have failed treatment with a biologic DMARD (bDMARD) [4–6]. Currently, 3 JAK inhibitors are approved for the treatment of RA in the USA, EU, and Japan: tofacitinib, baricitinib, and most recently upadacitinib. These treatments have shown efficacy and an acceptable safety profile in global populations in the phase 3 clinical trial programs, as well as in trials run specifically in Japanese patients with RA, although higher rates of herpes zoster have been reported in Japan and other Asian countries versus the global population [7–11].

Upadacitinib (ABT-494) is an oral JAK inhibitor engineered for greater selectivity toward JAK1 versus JAK2, JAK3, and TYK2 [12] and is approved at the dose of 15 mg once daily (QD) in the USA and EU, and at the doses of 7.5 mg QD and 15 mg QD in Japan. Upadacitinib was efficacious and well tolerated in both global phase 2 [13, 14] and phase 3 studies [15–18]. In the SELECT-SUNRISE study, upadacitinib in combination with csDMARDs improved outcomes at week 12 versus placebo in Japanese patients with an inadequate response to csDMARDs [11]. Here we report the results of the long-term extension phase of the SELECT-SUNRISE study, with efficacy and safety data up to week 84.

## Methods

### Study design and patients

SELECT-SUNRISE (NCT02720523) was a phase 2b/3, multicenter study that included 2 periods (Supplementary Figure 1 [11]). In period 1, Japanese patients with moderately to severely active RA and an inadequate response to csDMARDs, and on a stable dose of csDMARDs, were randomized to receive upadacitinib 7.5 mg, 15 mg, or 30 mg QD, or placebo for 12 weeks during a double-blind period. All patients who completed period 1 entered the blinded extension at 12 weeks, in which they continued with upadacitinib 7.5 mg, 15 mg, or 30 mg QD or were switched from placebo to upadacitinib 7.5 mg, 15 mg, or 30 mg QD according to prespecified randomization assignments.

In patients who failed to achieve Clinical Disease Activity Index (CDAI) ≤ 10 at week 24, the investigator was asked to adjust the patient’s background RA therapies, and initiation of or change in corticosteroids, nonsteroidal anti-inflammatory drugs, acetaminophen, or csDMARDs was allowed as per the local label. Starting at week 24, at least 20% improvement in both tender joint count and swollen joint count compared with baseline was required to remain in the study. Any patient who did not fulfill this criterion at 2 consecutive visits was discontinued from the study.

The study was conducted according to the International Conference on Harmonization of Technical Requirements for Pharmaceuticals for Human Use guidelines, applicable regulations and guidelines governing clinical study conduct, and the Declaration of Helsinki. Study-related documents were reviewed and approved by independent ethics committees and institutional review boards. All patients provided written informed consent. The list of Japan study sites is provided in Supplementary Text 1.

### Assessments

American College of Rheumatology (ACR) response rates (ACR20 [primary endpoint for the 12-week, double-blind, treatment period], ACR50, and ACR70), proportions of patients achieving low disease activity (LDA) and remission based on the Disease Activity Score of 28 joints with C-reactive protein (DAS28[CRP]) (LDA ≤ 3.2 and remission < 2.6), CDAI (LDA ≤ 10 and remission ≤ 2.8), Simplified Disease Activity Index (SDAI) (LDA ≤ 11 and remission ≤3.3), and measures of functional impairment (change from baseline in Health Assessment Questionnaire-Disability Index [HAQ-DI] and morning stiffness severity [numeric rating scale]) were assessed at baseline, weeks 1, 2, 4, 8, 12, 16, 20, 24, and every 12 weeks up to week 84. The proportion of patients reporting a minimal clinically important improvement in HAQ-DI (decrease from baseline of ≥ 0.22) was also assessed.

Treatment-emergent adverse events (TEAEs) and clinical laboratory testing that included hematology, chemistry, and urinalysis were recorded during the entire duration of the study. Laboratory data were processed at a central laboratory and categorized according to the Outcome Measures in Rheumatology criteria. For creatine phosphokinase (CPK) and serum creatinine, National Cancer Institute Common Toxicity Criteria (NCI-CTC) were used. Patients were asked to fast for a minimum of 8 h prior to providing blood samples for laboratory analysis.

### Statistical analysis

The full analysis set, which included all randomized patients who received at least 1 dose of study drug, was used for all efficacy analyses. The safety analysis set, which comprised all patients who received at least 1 dose of study drug, was used for the long-term safety analysis, with patients who switched from placebo included in their respective upadacitinib groups. Nonresponder imputation was used for binary endpoints, and as-observed data are shown for continuous endpoints. As-observed data for binary endpoints are shown in the Supplementary Information.

## Results

### Patients

**Fig. 1:**
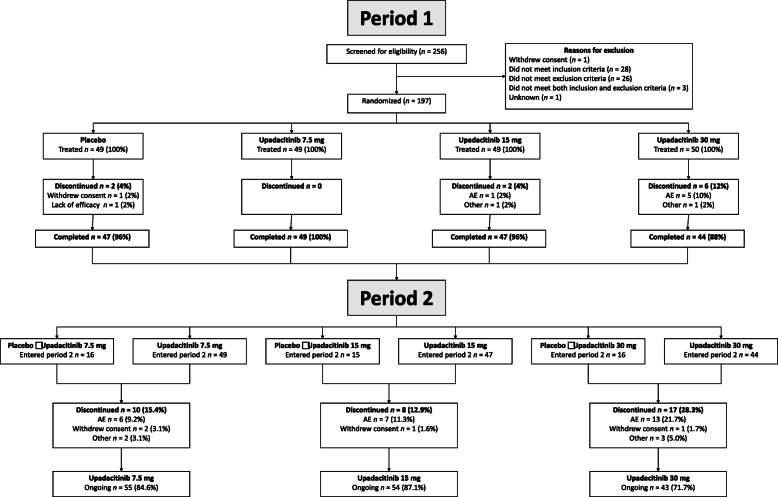
Patient disposition in periods 1 (12-week, placebo-controlled) and 2 (blinded extension; cut-off December 26, 2018). AE, adverse event

Of the 197 Japanese patients randomized in SELECT-SUNRISE, 187 (94.9%) completed 12 weeks on the study drug and entered the blinded extension period (Fig. 1). The cut-off date for this interim analysis was December 26, 2018, when all patients ongoing in the study had completed their week 84 visit. At the cut-off date, 152 (77.2%) patients were ongoing in period 2. More patients in the upadacitinib 30 mg group had withdrawn because of adverse events (AEs) compared with other treatment groups (Fig. 1).

Patient characteristics and baseline demographics were generally balanced across treatment arms (Supplementary Table 1 [11]).

### Efficacy outcomes

**Fig. 2:**
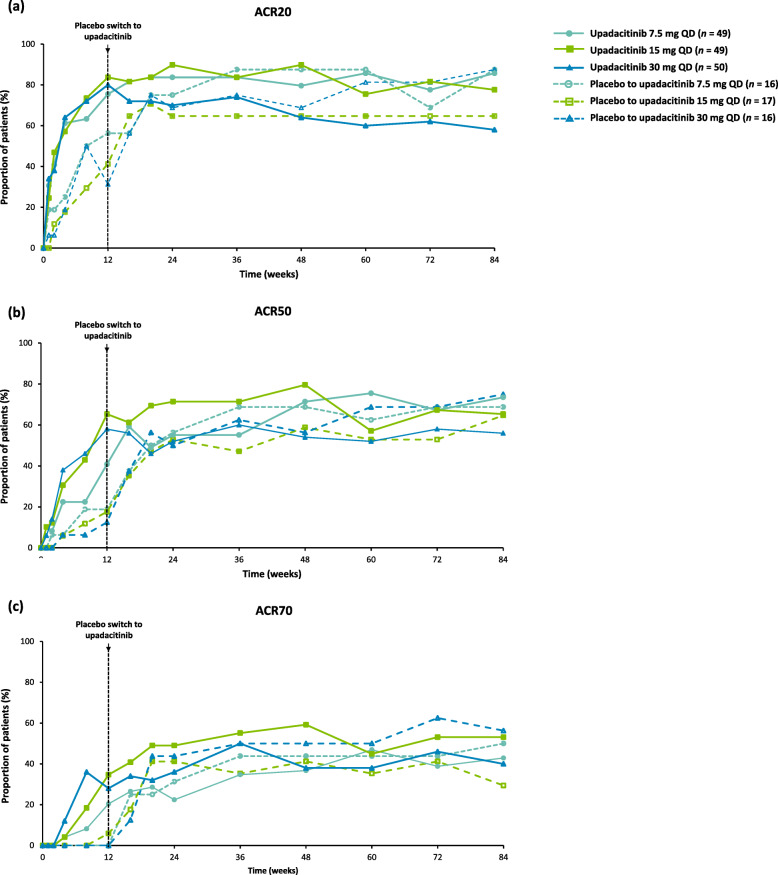
**a** ACR20, **b** ACR50, and **c** ACR70 responses over 84 weeks (full analysis set; NRI). ACR20/50/70, 20/50/70% improvement in American College of Rheumatology criteria; NRI, nonresponder imputation; QD, once daily

As reported previously, upadacitinib (all doses) met the primary endpoint of ACR20 response at week 12 in this population of Japanese patients with RA and an inadequate response to csDMARDs [11]. In patients originally randomized to upadacitinib, ACR20 response rates for patients on continuous upadacitinib (to week 84) generally showed continued improvement or maintenance over time through week 84 (Fig. 2a). In patients who were originally randomized to placebo, improvements in ACR20 response were seen following switch to upadacitinib at week 12 (Fig. 2a). ACR20 response rates at week 84 were 85.7%, 77.6%, and 58.0% for patients continuing upadacitinib 7.5 mg, 15 mg, and 30 mg, respectively, and were similar for patients who had switched to upadacitinib at week 12. Comparable trends were demonstrated for ACR50 and ACR70 responses, although the 15 mg and 30 mg doses of upadacitinib were associated with more rapid and numerically higher initial ACR50/70 responses compared with the 7.5 mg dose (Fig. 2b, c). Similar results were seen with as-observed data (Supplementary Figure 2).

**Fig. 3:**
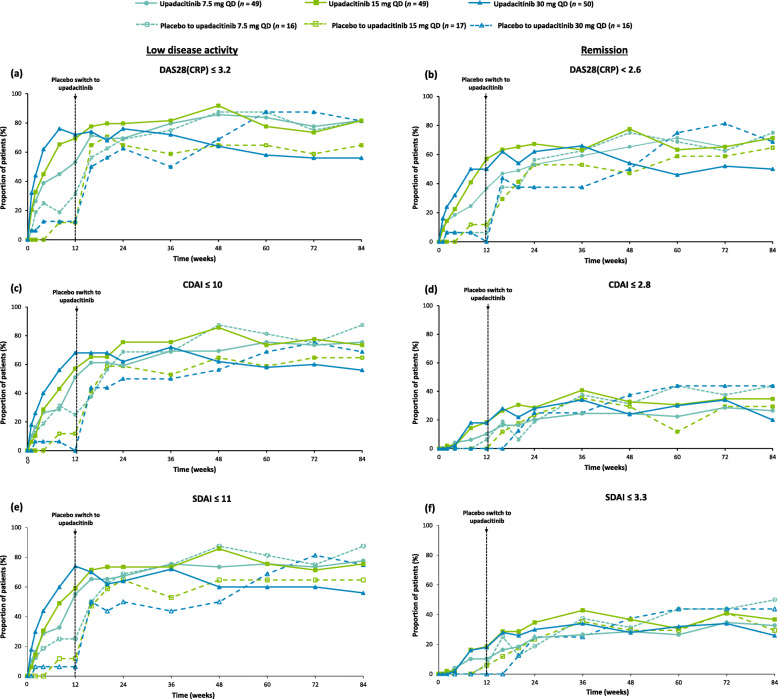
Proportions of patients achieving low disease activity and remission over 84 weeks, respectively based on **a**, **b** DAS28(CRP) (≤ 3.2 and < 2.6), **c**, **d** CDAI (≤ 10 and ≤ 2.8), and **e**, **f** SDAI (≤ 11 and ≤ 3.3) (full analysis set; NRI). CDAI, Clinical Disease Activity Index; DAS28(CRP), Disease Activity Score of 28 joints with C-reactive protein; NRI, nonresponder imputation; QD, once daily; SDAI, Simplified Disease Activity Index

Rates of LDA and remission defined by DAS28(CRP), which were achieved over 12 weeks in patients originally randomized to upadacitinib, improved or were maintained at week 84 (Fig. 3a and b). The proportion of patients achieving these endpoints also improved in patients originally randomized to placebo following switch to upadacitinib at week 12 (Fig. 3a and b). Comparable trends were demonstrated for rates of LDA and remission defined by CDAI and SDAI (Fig. 3c–f), and were similar with as-observed data (Supplementary Figure 3).

**Fig. 4:**
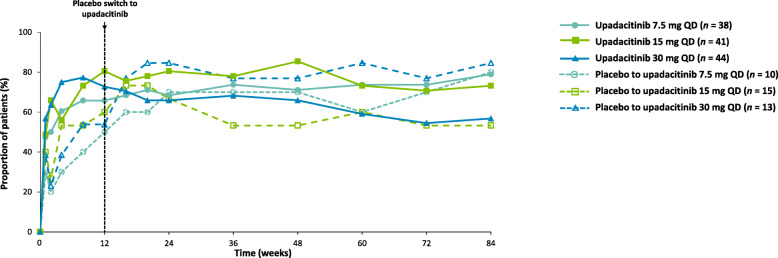
Proportions of patients reporting minimal clinically important improvement in Health Assessment Questionnaire-Disability Index (HAQ-DI decrease from baseline of ≥ 0.22) over 84 weeks (full analysis set; NRI). NRI, nonresponder imputation; QD, once daily

The proportion of patients reporting a minimal clinically important improvement in HAQ-DI (decrease from baseline of ≥ 0.22) increased over 84 weeks with upadacitinib (Fig. 4), with similar results using as-observed data (Supplementary Figure 4a). Improvements were also seen in change from baseline in HAQ-DI and severity of morning stiffness, although smaller improvements were observed in patients who switched from placebo to upadacitinib 7.5 mg compared with those in other treatment groups (Supplementary Figure 4b, c).

### Safety

The rate of overall TEAEs, serious TEAEs, and TEAEs leading to discontinuation tended to show a numerically dose-dependent increase, with slightly higher rates in the 15 mg group versus the 7.5 mg group, and the highest rates observed in the 30 mg group (Table 1). No deaths were reported in the upadacitinib 7.5 mg or 15 mg groups. Two deaths, including 1 treatment-emergent death and 1 non-treatment-emergent death, were reported in the 30 mg group. The treatment-emergent death occurred on day 384 and was due to respiratory failure in a 65-year-old female who experienced a pneumothorax while taking a bath (adjudicated as a cardiovascular death). The non-treatment-emergent death was due to aortic aneurysm rupture on post-study day 131 in a 66-year-old female; this patient had received the study drug for a total of 14 days which had been discontinued for an event of interstitial pneumonia.

**Table 1:** Summary of treatment-emergent adverse events incidence rate per 100 patient-years in patients who received upadacitinib over 84 weeks (safety analysis set)

AE, incidence (*n*/100 PY)	Upadacitinib 7.5 mg QD (***n*** = 65)	Upadacitinib 15 mg QD (***n*** = 64)	Upadacitinib 30 mg QD (***n*** = 66)
AEs	64 (316.8)	64 (369.9)	66 (458.3)
Serious AEs	13 (10.8)	17 (15.3)	20 (20.3)
AEs leading to discontinuation of study drug	6 (4.6)	8 (6.4)	20 (18.6)
Deaths^†^	0	0	2 (1.8)
Infection	52 (117.1)	57 (140.0)	58 (167.1)
Serious infection	6 (4.7)	8 (6.7)	13 (12.7)
Opportunistic infection	1 (0.8)	4 (3.3)	8 (7.6)
Herpes zoster	9 (7.3)	14 (12.3)	16 (16.5)
Active/latent tuberculosis	0	0	1 (0.9)
Malignancy (incl. NMSC)	0	1 (0.8)	1 (0.9)
Hepatic disorder	7 (5.8)	7 (6.1)	8 (8.1)
Gastrointestinal perforation	0	1 (0.8)	1 (0.9)
MACE^‡^	1 (0.8)	0	1 (0.9)
Adjudicated VTE	0	0	1 (0.9)
Anemia	0	2 (1.6)	5 (4.9)
Neutropenia	1 (0.8)	2 (1.7)	7 (7.1)
Lymphopenia	5 (3.9)	7 (5.9)	8 (7.8)
CPK elevation	5 (4.0)	7 (6.1)	8 (8.2)
Renal dysfunction	0	0	1 (0.9)

Two subjects treated with placebo in period 1 discontinued treatment and thus were not included in the safety analysis set

*AE* adverse event, *CPK* creatine phosphokinase, *MACE*, major adverse cardiovascular event, *NMSC* nonmelanoma skin cancer, *PY* patient-years, *QD* once daily, *VTE*, venous thromboembolism

^†^Includes non-treatment-emergent deaths

^‡^Defined as cardiovascular death, nonfatal myocardial infarction, and nonfatal stroke

The most frequently reported TEAE was nasopharyngitis. The rates of treatment-emergent serious infections (incidence rates 4.7, 6.7, and 12.7 per 100 patient-years, respectively), opportunistic infections (incidence rates 0.8, 3.3, and 7.6 per 100 patient-years, respectively), and herpes zoster (incidence rates 7.3, 12.3, and 16.5 per 100 patient-years, respectively) appeared to be dose dependent, with lower rates observed with upadacitinib 7.5 mg and 15 mg versus 30 mg (Table 1). The most frequently reported serious infections (with upadacitinib 7.5 mg, 15 mg, and 30 mg, respectively) were herpes zoster (incidence rates 2.3, 1.7, and 3.9 per 100 patient-years), pneumonia (incidence rates 1.6, 0, and 2.0 per 100 patient-years), and *Pneumocystis jirovecii* pneumonia (incidence rates 0, 0, and 3.9 per 100 patient-years). *Pneumocystis jirovecii* pneumonia, only observed in the upadacitinib 30 mg group (4 events), was the most commonly reported opportunistic infection. The majority of the herpes zoster events were nonserious and involved only 1 dermatome. Serious herpes zoster events included 3 events in the upadacitinib 7.5 mg group, 2 events in the upadacitinib 15 mg group, and 4 events in the upadacitinib 30 mg group. No herpes zoster event was reported as having central nervous system involvement, and there was 1 case of serious herpes zoster with ophthalmic involvement in the 30 mg group. Six patients (3 each in the 15 mg and 30 mg groups, incidence rates 2.4 and 2.8 per 100 patient-years, respectively) reported disseminated herpes zoster infection (3 serious, 3 nonserious), including 5 disseminated cutaneous herpes zoster events and 1 disseminated noncutaneous herpes zoster event in the oral cavity. Of patients with an event of herpes zoster, none had a herpes zoster vaccination prior to baseline (herpes zoster vaccination prior to baseline was unknown for 2 subjects), 28 (71.8%) were ≥ 50 years of age, and 3 (7.7%) had a prior history of herpes zoster. There was no active tuberculosis reported.

Two malignancies were reported: acute lymphocytic leukemia in the upadacitinib 15 mg group and Hodgkin disease in the upadacitinib 30 mg group. The subject with acute lymphocytic leukemia was taking concomitant methotrexate and no particular risk factor was identified for the subject with Hodgkin disease. Two patients had TEAEs of gastrointestinal perforation (1 patient with intestinal perforation in the 15 mg group and 1 with events of anal fistula and perirectal abscess in the 30 mg group). Four adjudicated cardiovascular events were reported: 2 major adverse cardiovascular events (respiratory failure adjudicated to cardiovascular death with upadacitinib 30 mg, and cerebral infarction adjudicated as a nonfatal stroke with upadacitinib 7.5 mg), 1 transient ischemic attack (upadacitinib 7.5 mg), and 1 deep vein thrombosis (upadacitinib 30 mg). All patients, except for the patient with respiratory failure, had cardiovascular risk factors at study entry. With the exception of deep vein thrombosis, all events were reported as serious TEAEs and as having a reasonable possibility of being related to the study drug.

Rates of neutropenia, lymphopenia, anemia, and CPK elevation appeared to be dose dependent; rates were slightly higher in the 15 mg group versus the 7.5 mg group and were highest in the 30 mg group (Table 1). No patient discontinued the study drug due to a TEAE of anemia or neutropenia. Two patients, both receiving upadacitinib 30 mg, had TEAEs of lymphopenia that led to discontinuation of the study drug. One patient receiving upadacitinib 15 mg discontinued the study drug due to an asymptomatic TEAE of CPK elevation. No patient had rhabdomyolysis. The rate of hepatic disorders was comparable across the doses, and renal dysfunction was uncommon.

The proportion of patients with grade 3 decreases in hemoglobin and lymphocyte levels was higher with upadacitinib 30 mg compared with the other doses (Table 2). However, over half of patients randomized to upadacitinib treatment had grade 2 or 3 lymphocyte counts at baseline, with the highest rates in the upadacitinib 30 mg group (Table 3). Of the grade 3 or 4 decreases in hemoglobin, none led to study drug discontinuation, and approximately half were isolated events, occurring at only 1 time point during the treatment period. Of 10 patients with grade 4 lymphocyte decreases, 3 discontinued the study drug and 3 had infectious events (1 event each of pneumonia, infectious enteritis, and *Pneumocystis jirovecii* pneumonia) around the onset of lymphopenia (approximately − 16 to 5 days). Grade 3 or 4 changes in neutrophils, leukocytes, alanine aminotransferase (ALT), aspartate aminotransferase (AST), and CPK were rare. Increases in ALT and AST were mostly isolated events, and all returned to normal during the treatment period. One patient (upadacitinib 7.5 mg) had a grade 4 decrease in neutrophil levels; this patient had nasopharyngitis around the time of neutropenia and did not discontinue the study drug due to a decreased neutrophil count. One patient who received upadacitinib 15 mg discontinued the study drug during the study follow-up period due to an asymptomatic increase in blood CPK. No patient had a grade 3 or 4 increase in serum creatinine.

**Table 2:** Patients with worsening in grade severity (grades 3 or 4) for laboratory parameters at any time during 84 weeks, including single isolated values (safety analysis set)

Lab parameter^†^ *n* (%)	Upadacitinib 7.5 mg QD (***n*** = 65)	Upadacitinib 15 mg QD (***n*** = 64)	Upadacitinib 30 mg QD (***n*** = 66)
Hemoglobin (g/L)			
Grade 3 (70 to < 80 or ↓ 21 to < 30)	0	1 (1.6)	9 (13.6)
Grade 4 (< 70 or ↓ ≥ 30)	1 (1.5)	0	4 (6.1)
Lymphocytes (× 10^9^/L)			
Grade 3 (0.5 to < 1.0)	30 (46.2)	27 (42.2)	36 (54.5)
Grade 4 (< 0.5)	4 (6.2)	2 (3.1)	4 (6.1)
Neutrophils (× 10^9^/L)			
Grade 3 (0.5 to < 1.0)	1 (1.5)	1 (1.6)	2 (3.0)
Grade 4 (< 0.5)	1 (1.5)	0	0
Leukocytes (× 10^9^/L)			
Grade 3 (1.0 to < 2.0)	1 (1.5)	0	1 (1.5)
Grade 4 (< 1.0)	0	0	0
ALT (U/L)			
Grade 3 (3.0–8.0 × ULN)	1 (1.5)	3 (4.7)	2 (3.0)
Grade 4 (> 8.0 × ULN)	0	0	1 (1.5)
AST (U/L)			
Grade 3 (3.0–8.0 × ULN)	1 (1.5)	2 (3.1)	2 (3.0)
Grade 4 (> 8.0 × ULN)	0	0	0
CPK (U/L)			
Grade 3 (> 5–10 × ULN)	2 (3.1)	2 (3.1)	3 (4.5)
Grade 4 (> 10 × ULN)	0	1 (1.6)	4 (6.1)
Creatinine (μmol/L)			
Grade 3 (> 3.0–6.0 × ULN)	0	0	0
Grade 4 (> 6.0 × ULN)	0	0	0

Two subjects treated with placebo in period 1 discontinued treatment and thus were not included in the safety analysis set. Notes: For CPK and creatinine, NCI-CTC criteria were used. Post-baseline grade must be higher than the baseline grade

*ALT* alanine aminotransferase, *AST* aspartate aminotransferase, *CPK* creatine phosphokinase, *NCI-CTC* National Cancer Institute Common Toxicity Criteria, *OMERACT* Outcome Measures in Rheumatology, *QD* once daily, *ULN* upper limit of normal

^†^The toxicity grading is based on OMERACT criteria (Rheumatology Common Toxicity Criteria v.2.0)

**Table 3:** Lymphocyte count as baseline (safety analysis set)

Lymphocytes^†^, × 10^9^/L, ***n*** (%)	Upadacitinib 7.5 mg QD (***n*** = 65)	Upadacitinib 15 mg QD (***n*** = 64)	Upadacitinib 30 mg QD (***n*** = 66)
Grade 0 (> 2.0)	5 (7.7)	9 (14.1)	7 (10.6)
Grade 1 (1.5 to < 2.0)	21 (32.3)	11 (17.2)	11 (16.7)
Grade 2 (1.0 to < 1.5)	33 (50.8)	31 (48.4)	37 (56.1)
Grade 3 (0.5 to < 1.0)	6 (9.2)	13 (20.3)	11 (16.7)

Two subjects treated with placebo in period 1 discontinued treatment and thus were not included in the safety analysis set

*QD* once daily

^†^The toxicity grading is based on Outcome Measures in Rheumatology criteria (Rheumatology Common Toxicity Criteria v.2.0)

## Discussion

The SELECT-SUNRISE study assessed the efficacy and safety profile of 3 doses of upadacitinib (7.5 mg, 15 mg, and 30 mg) in Japanese patients who had moderately to severely active RA despite a stable dose of csDMARDs. In the 12-week study, upadacitinib showed consistent improvement in clinical and patient-reported outcomes versus placebo across all 3 doses. These improvements increased or were maintained through to week 84 in the long-term extension in patients who were originally randomized to upadacitinib. In addition, patients who switched from placebo to upadacitinib at week 12 showed efficacy improvements up to week 84 similar to those observed for patients originally randomized to upadacitinib. This suggests that upadacitinib may be a favorable long-term option for Japanese patients with RA who have an inadequate response to csDMARDs.

Long-term efficacy was generally comparable across the 3 doses, although the 15 mg and 30 mg doses were associated with more rapid and overall numerically higher initial responses for more stringent measures of response (ACR70) and remission (defined by DAS28[CRP], CDAI, SDAI) compared with the 7.5 mg dose. A similar trend was observed in physical function (HAQ-DI; weeks 12–84) between groups switching from placebo. For all efficacy outcomes measured, the 30 mg dose provided no additional benefit compared with the 15 mg dose, suggesting that the 15 mg dose was associated with optimal efficacy. These results are consistent with the SELECT-EARLY Japanese sub-analysis, in which significant improvements were observed across primary (including radiographic) and secondary endpoints with upadacitinib 15 mg and 30 mg monotherapy versus methotrexate in methotrexate-naïve patients, with no additional benefits with the 30 mg dose, while the 7.5 mg dose led to significant improvements in the nonradiographic primary endpoint (ACR20) and secondary endpoints only [19].

Similar to the 12-week data [11], long-term safety data tended to show numerically dose-dependent increases in incidence rates of AEs, including serious AEs, serious infection, herpes zoster, and AEs leading to discontinuation of the study drug. Due to the small sample size, these data should be interpreted with caution.

Both the 7.5 mg and 15 mg doses of upadacitinib have been approved for the treatment of RA in Japan; however, efficacy and safety results from this study indicate upadacitinib 15 mg as the optimal dose for long-term treatment in these patients. Findings are consistent with the SELECT-EARLY Japanese sub-analysis, in which upadacitinib 15 mg was identified as the optimal dose versus the 7.5 mg and 30 mg doses. Of note, the efficacy and safety profile of upadacitinib in this study were broadly similar to those reported in global trials of upadacitinib; however, rates of herpes zoster were higher in this study versus global trials [15–18]. Higher rates of herpes zoster have similarly been reported in Japanese and Korean patients treated with baricitinib and tofacitinib compared with other geographical regions, although the reasons for this are unclear [9]. Similar to the overall rate of herpes zoster, the incidence of disseminated herpes zoster (3 cases each in the 15 mg and 30 mg groups, incidence rates 2.4 and 2.8 per 100 patient-years, respectively) tended to be numerically higher in this study compared with a global population (incidence rate < 0.1 per 100 patient-years with upadacitinib 15 mg [data on file]).

This study has several limitations, in common with all long-term studies. Efficacy data should be interpreted with caution, as there was no placebo comparison from week 12 onwards (although blinding was maintained) and no statistical comparisons were performed between upadacitinib treatment groups. Finally, changes in background medication were permitted from week 24 onwards, which may confound the efficacy and safety findings with upadacitinib.

## Conclusions

Overall, results demonstrate that upadacitinib is an efficacious and well-tolerated treatment option for Japanese patients with RA and an inadequate response to csDMARDs. Upadacitinib at the 15 mg dose showed the most favorable long-term benefit–risk profile in this Japanese population.

## Supplementary Information

**Additional file 1. Supplementary Figure 1. **

**Additional file 2. Supplementary Text 1.**

**Additional file 3. Supplementary Table 1**.

**Additional file 4. Supplementary Figure 2**.

**Additional file 5. Supplementary Figure 3.**

**Additional file 6. Supplementary Figure 4.**

## Data Availability

These clinical trial data can be requested by any qualified researchers who engage in rigorous, independent scientific research, and will be provided following review and approval of a research proposal and Statistical Analysis Plan (SAP) and execution of a Data Sharing Agreement (DSA). Data requests can be submitted at any time and the data will be accessible for 12 months, with possible extensions considered. For more information on the process, or to submit a request, visit the following link: https://www.abbvie.com/our-science/clinical-trials/clinical-trials-data-and-information-sharing/data-and-information-sharing-with-qualified-researchers.html.

## Abbreviations

ACR20/50/70: 20%/50%/70% improvement in American College of Rheumatology criteria
AE: Adverse event
ALT: Alanine transaminase
AST: Aspartate transaminase
bDMARD: Biologic disease-modifying anti-rheumatic drug
CDAI: Clinical Disease Activity Index
CPK: Creatine phosphokinase
csDMARD: Conventional synthetic disease-modifying anti-rheumatic drug
DAS28(CRP): Disease Activity Score of 28 joints with C-reactive protein
HAQ-DI: Health Assessment Questionnaire-Disability Index
JAK: Janus kinase
LDA: Low disease activity
MACE: Major adverse cardiovascular event
NMSC: Nonmelanoma skin cancer
NCI-CTC: National Cancer Institute Common Toxicity Criteria
NRI: Nonresponder imputation
OMERACT: Outcome Measures in Rheumatology
QD: Once daily
RA: Rheumatoid arthritis
SDAI: Simplified Disease Activity Index
TEAE: Treatment-emergent adverse event
TYK2: Tyrosine kinase 2
ULN: Upper limit of normal
VTE: Venous thromboembolism
