# Supplementary figures and images for: Efficacy and safety of upadacitinib over 84 weeks in Japanese patients with rheumatoid arthritis (SELECT-SUNRISE)
Source: Arthritis Res Ther. 2021 Jan 6;23:9. doi: 10.1186/s13075-020-02387-6 (PMC7789301; doi:10.1186/s13075-020-02387-6)

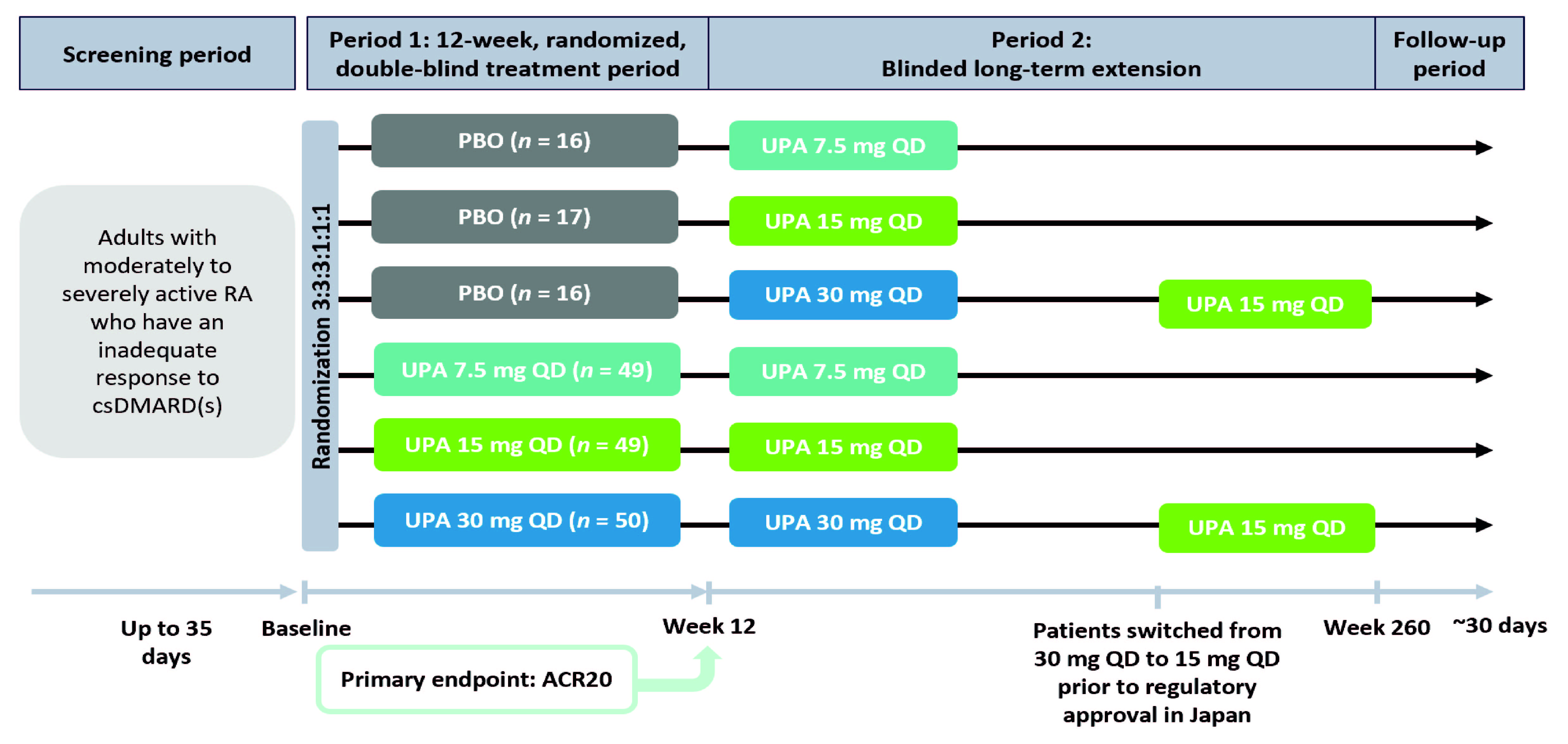

Supplement: Supplementary file 1 — Additional file 1. Supplementary Figure 1. [file 13075_2020_2387_MOESM1_ESM.jpg]

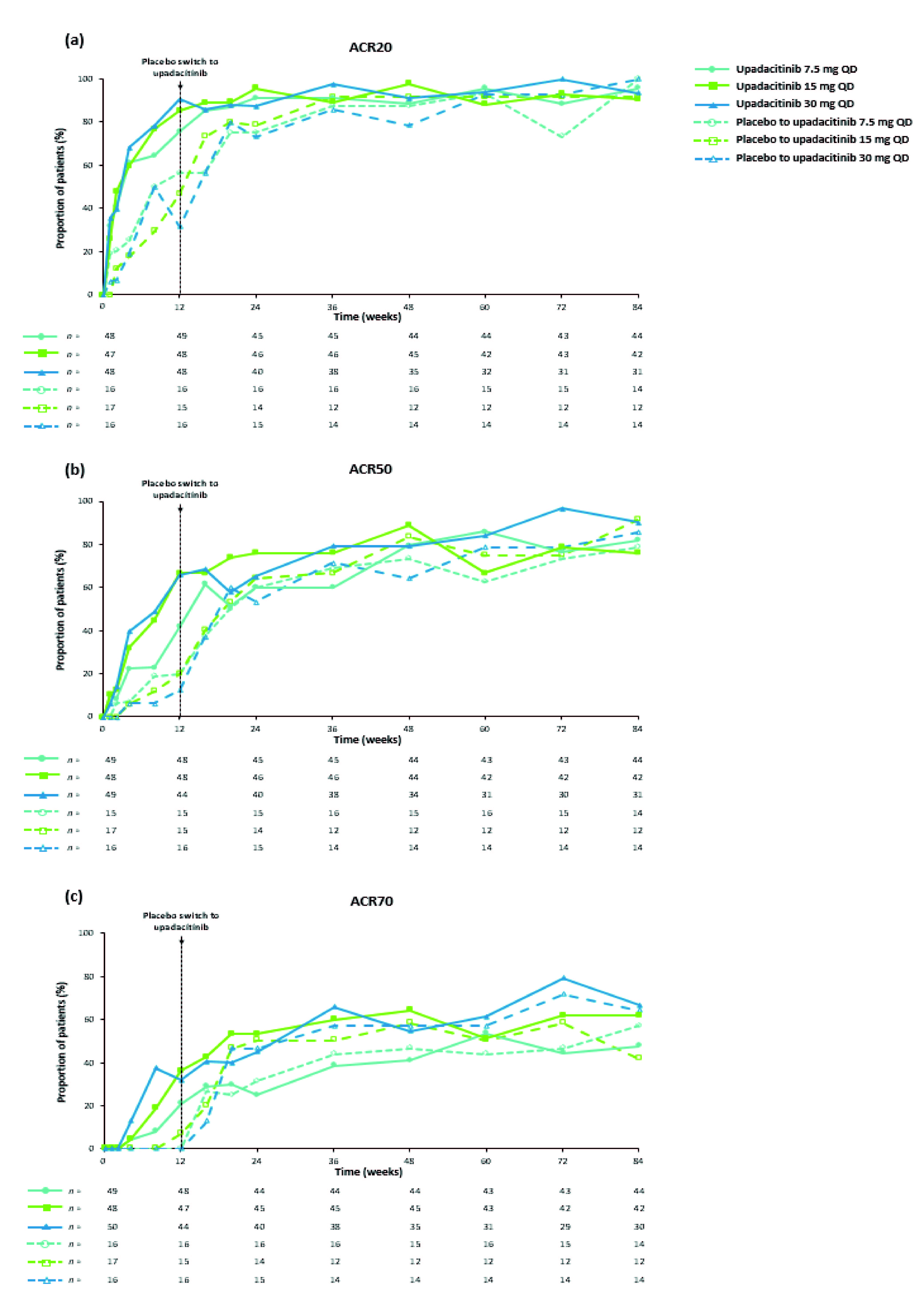

Supplement: Supplementary file 4 — Additional file 4. Supplementary Figure 2. [file 13075_2020_2387_MOESM4_ESM.jpg]

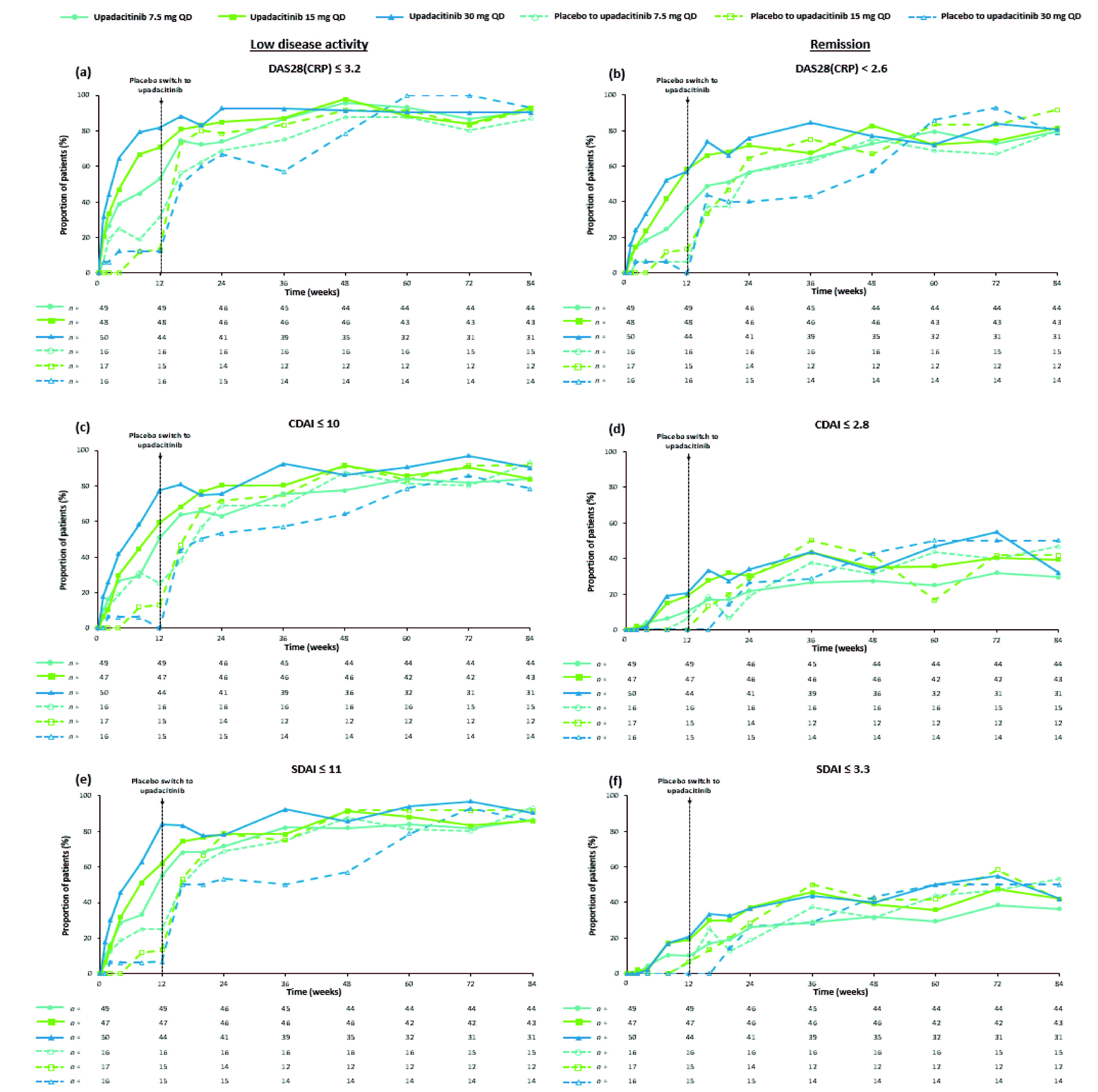

Supplement: Supplementary file 5 — Additional file 5. Supplementary Figure 3. [file 13075_2020_2387_MOESM5_ESM.jpg]

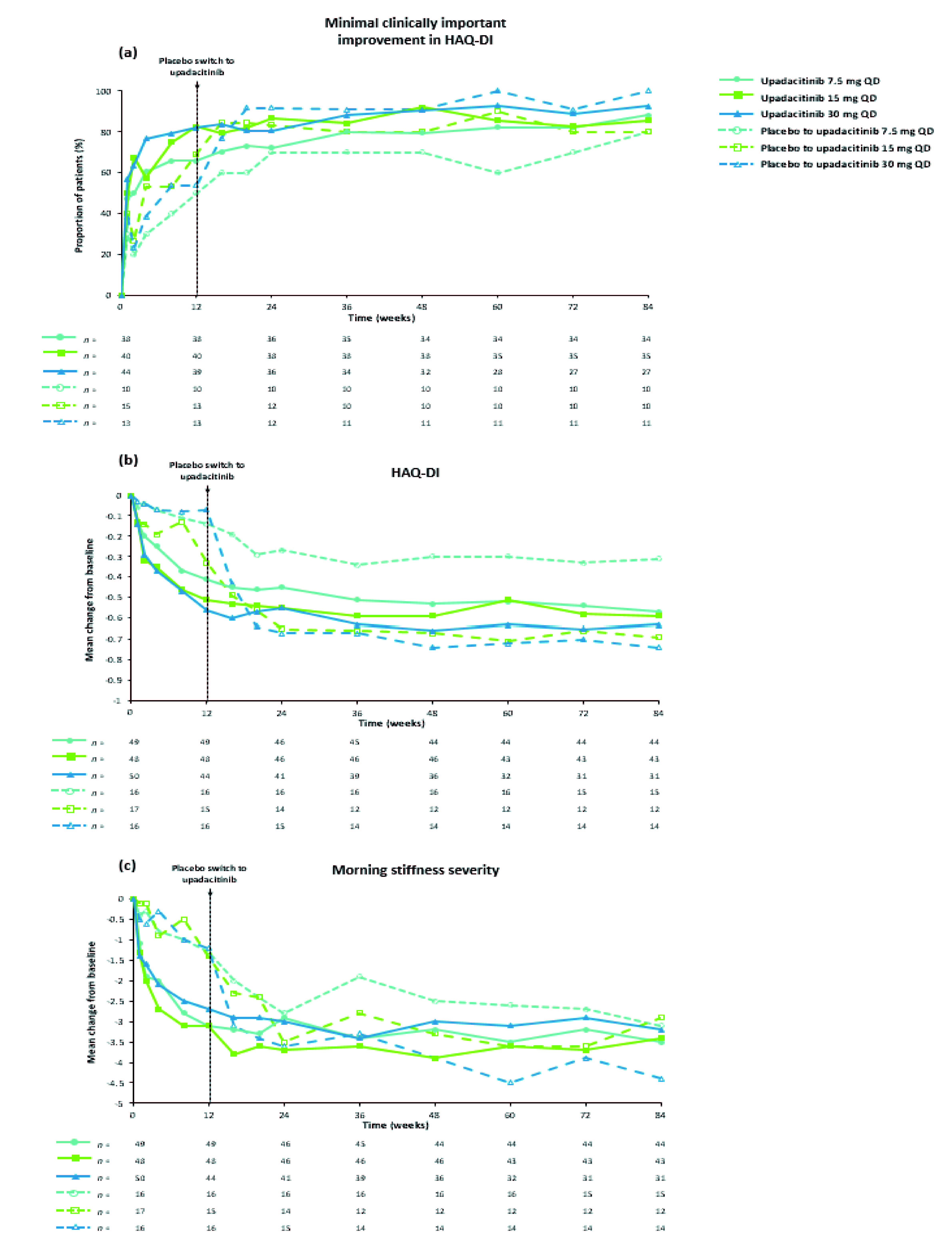

Supplement: Supplementary file 6 — Additional file 6. Supplementary Figure 4. [file 13075_2020_2387_MOESM6_ESM.jpg]
