# Supplementary material for: Efficacy and safety of upadacitinib over 84 weeks in Japanese patients with rheumatoid arthritis (SELECT-SUNRISE)
Source: Arthritis Res Ther. 2021 Jan 6;23:9. doi: 10.1186/s13075-020-02387-6 (PMC7789301; doi:10.1186/s13075-020-02387-6)
Supplement: Supplementary file 2 — Additional file 2. Supplementary Text 1. [file 13075_2020_2387_MOESM2_ESM.docx]

## Additional File 2: Supplementary Text 1. List of SELECT-SUNRISE Japan study sites.

This study was conducted at the following sites: Sapporo City General Hospital, Shono Rheumatism Clinic, Kumamoto Orthopedic Hospital, Marunouchi Hospital, Jichi Medical University Hospital, Inoue Hospital, Saitama Medical Center, St. Luke's International Hospital, Matsubara Mayflower Hospital, Hospital of the University of Occupational and Environmental Health, Keio University Hospital, Hiroshima Rheumatology Clinic, Nagaoka Red Cross Hospital, Medical Corporation Keiai Kai Clinic, Tokito Clinic Rheumatology and Orthopedics Surgery, St. Mary's Hospital, Katayama Orthopedic Rheumatology Clinic, Hyogo College of Medicine College Hospital, Nihon University Itabashi Hospital, NHO Osaka Minami Med Ctr, Yu Family Clinic, National Hospital Organization Sagamihara National Hospital, Takaoka Rheumatic Orthopedic Clinic (formerly Honjo Rheumatism Clinic), Sagawa Akira Rheumatology Clin, Kondo Clinic for Ortho & Rheum, Sugimoto Rheumatology and Internal Medicine Clinic, Oki Medical Clinic, Hamanomachi Hospital, Miyashita Rheumatology Clinic, Okayama City Gen Med Ctr, Ichinomiya Municipal Hospital, JP Red Cross Nagoya Daiichi, Hikarigaoka Spellman Hospital, National Hospital Organization Asahikawa Medical Center, NHO Chiba-East-Hospital, National Hospital Organization Tokyo Medical Center, National Hospital Organization Osaka Toneyama Medical Center, Setagaya Rheumatic Clinic, Kagawa University Hospital, Tokyo Women’s Medical University Hospital, Toho University Ohashi Medical Center, Kyushu University Hospital, Niigata Rheumatic Center, Nagoya University Hospital, Medical Corporation Uchida Clinic, Oribe Clinic of Rheumatology and Internal Medicine, Takikawa Municipal Hospital, and Tohoku University Hospital.
