# Supplementary material for: Efficacy and safety of upadacitinib over 84 weeks in Japanese patients with rheumatoid arthritis (SELECT-SUNRISE)
Source: Arthritis Res Ther. 2021 Jan 6;23:9. doi: 10.1186/s13075-020-02387-6 (PMC7789301; doi:10.1186/s13075-020-02387-6)
Supplement: Supplementary file 3 — Additional file 3. Supplementary Table 1. [file 13075_2020_2387_MOESM3_ESM.docx]

## Additional File 3: Supplementary Table 1. Demographics and baseline characteristics (reproduced from ([11](#_ENREF_11))])

| Parameter^*^ | Placebo  (***n*** = 49) | Upadacitinib  7.5 mg QD  (***n*** = 49) | Upadacitinib  15 mg QD  (***n*** = 49) | Upadacitinib  30 mg QD  (***n*** = 50) |
| --- | --- | --- | --- | --- |
| **Demographic characteristics** |  |  |  |  |
| Age (years) | 54.3 (13.0) | 55.8 (11.0) | 56.0 (12.5) | 54.7 (12.2) |
| Female, *n* (%) | 42 (85.7) | 34 (69.4) | 36 (73.5) | 43 (86.0) |
| Weight (kg) | 56.5 (13.7) | 59.2 (10.7) | 58.6 (10.1) | 56.9 (13.0) |
| BMI (kg/m^2^) | 22.8 (4.5) | 23.0 (2.7) | 23.2 (3.4) | 23.1 (5.0) |
| Time since RA diagnosis (years), median (range) | 2.1 (0.4–19.2) | 4.0 (0.4–31.3) | 2.9 (0.4–34.7) | 2.8 (0.3–16.3) |
| RF positive, *n* (%) | 31 (63.3) | 38 (77.6) | 36 (73.5) | 33 (66.0) |
| Anti-CCP positive, *n* (%) | 40 (81.6) | 42 (85.7) | 38 (77.6) | 46 (92.0) |
| Prior bDMARD exposure, *n* (%) | 3 (6.1) | 5 (10.2) | 6 (12.2) | 3 (6.0) |
| **csDMARD use at baseline, *n* (%)**  MTX alone  MTX plus other csDMARD  csDMARD other than MTX | 29 (59.2)  14 (28.6)  6 (12.2) | 25 (51.0)  13 (26.5)  11 (22.4) | 28 (57.1)  12 (24.5)  9 (18.4) | 37 (74.0)  7 (14.0)  6 (12.0) |
| MTX dose (mg/week)^†^ | 10.1 (2.5) | 10.3 (2.6) | 9.2 (1.9) | 10.0 (2.3) |
| Oral steroid use, *n* (%) | 24 (49.0) | 26 (53.1) | 28 (57.1) | 24 (48.0) |
| Oral glucocorticoid dose (mg/day)^‡^ (prednisone equivalent dose) | 3.8 (2.1) | 3.3 (1.4) | 3.8 (1.9) | 3.6 (1.3) |
| **Disease characteristics** |  |  |  |  |
| TJC68 | 16.8 (11.4) | 16.3 (8.9) | 17.8 (12.6) | 16.3 (10.8) |
| SJC66 | 10.9 (4.7) | 11.7 (4.9) | 14.0 (7.8) | 11.7 (5.3) |
| hsCRP (mg/L), median (range) | 9.6 (1.3–103.0) | 8.3 (1.0–47.5) | 7.8 (0.8–84.6) | 7.0 (1.2–51.1) |
| DAS28(CRP) | 5.2 (0.8) | 5.1 (0.8) | 5.1 (1.1) | 5.0 (0.9) |
| CDAI | 31.0 (9.9) | 29.1 (8.1) | 32.1 (12.0) | 29.8 (10.7) |
| SDAI | 32.8 (10.1) | 30.4 (8.3) | 33.7 (12.8) | 31.0 (11.1) |
| HAQ-DI | 1.0 (0.7) | 0.9 (0.7) | 1.0 (0.7) | 0.9 (0.6) |
| FACIT-F | 35.2 (8.1) | 34.6 (9.6) | 33.8 (11.2) | 36.6 (9.2) |
| SF-36 PCS | 39.0 (7.1) | 40.0 (6.7) | 40.5 (7.9) | 42.4 (6.3) |
| RA-WIS | 10.6 (6.3) | 10.6 (6.1) | 9.5 (6.4) | 8.0 (6.0) |
| Morning stiffness severity | 4.6 (2.7) | 4.8 (2.6) | 4.9 (2.9) | 3.8 (2.8) |
| Morning stiffness duration (minutes), median (range) | 60.0 (0–1440.0) | 60.0 (0–540.0) | 50.0 (0–1440.0) | 30.0 (0–1440.0) |
| *Abbreviations: bDMARD* biologic disease-modifying anti-rheumatic drug, *BMI* body mass index, *CCP* cyclic citrullinated peptide, *csDMARD* conventional synthetic disease-modifying anti-rheumatic drug; *MTX* methotrexate, *hsCRP* high-sensitivity C-reactive protein, *DAS28(CRP)* Disease Activity Score of 28 joints with C-reactive protein, *CDAI* Clinical Disease Activity Index, *HAQ-DI* Health Assessment Questionnaire-Disability Index, *FACIT-F* Functional Assessment of Chronic Illness Therapy-Fatigue, *QD* once daily, *RA-WIS* Rheumatoid Arthritis-Work Instability Scale, *RF*, rheumatoid factor; *SD* standard deviation; *SDAI* Simplified Disease Activity Index, *SF-36 PCS* Short Form (36-item) Physical Component Summary, *SJC66* swollen joint count of 66 joints, *TJC68*, tender joint count of 68 joints  ^*^Data are mean (SD) unless otherwise indicated ^†^Mean MTX dose calculated only for patients receiving MTX ^‡^Mean glucocorticoid dose calculated only for patients receiving glucocorticoids | | | | |
